# Domestic firearm violence against women (2018–2021)^☆^

**DOI:** 10.1016/j.sopen.2024.01.010

**Published:** 2024-01-15

**Authors:** Jonathan Shipley, Megan Donnelly, Catherine Kuza, Areg Grigorian, Lourdes Swentek, Theresa Chin, Nolan Brown, Ninh Nguyen, Jeffry Nahmias

**Affiliations:** a University of California, Irvine, Department of Surgery, Division of Trauma, Burns and Surgical Critical Care, Orange, CA, USA; b Keck School of Medicine of the University of Southern California, Department of Anesthesia, Los Angeles, CA, USA

**Keywords:** Domestic firearm violence, COVID-19, Firearm violence against women, Domestic violence

## Abstract

**Background:**

Over 50 % of US female homicides occur during domestic violence, with half involving firearms. Public health measures to control COVID-19 may have isolated individuals with abusive partners at a time when firearm sales and new firearm ownership surged. This study sought to evaluate trends in domestic firearm violence (DFV) over time, hypothesizing that rates of DFV increased in the wake of COVID-19.

**Materials and methods:**

A retrospective query of the Gun Violence Archive (2018–2021) was conducted for incidents of DFV. The primary outcome was the number of DFV-related shootings. Statistical testing, including one-way and two-way ANOVAs, was performed to compare monthly rates of DFV over time and to compare DFV per 100,000 women in states with strong versus weak gun laws.

**Results:**

Average monthly DFV incidents rose nationwide during this study's time period, though injuries and fatalities did not. States with weaker gun laws had increased incidents, deaths, and injuries from 2018 to 2021 (all p<0.05). In a two-way ANOVA, stronger gun laws were associated with fewer incidents of DFV when compared with weaker gun law states. We also found that the use of a long gun in DFV more often resulted in a victim's death when compared to a handgun (p<0.01).

**Conclusion:**

DFV incidents increased over time. States with weaker gun laws bore the brunt of the violence, demonstrating that DFV may be curtailed through legislative efforts. Methods of injury prevention aimed at preventing and reducing domestic violence and improving firearm safety may curtail DFV.

## Introduction

The coronavirus disease 2019 (COVID-19) has become one of the deadliest pandemics in the modern era [1]. Both the pandemic itself and the public health measures implemented to reduce disease transmission have had substantial global consequences [1]. For instance, in the United States (US), mandatory universal masking, closure of schools and businesses, and stay-at-home (SAH) orders have significantly disrupted everyday life for the average American [2,3]. Moreover, not every American has been affected equally. Prior research results from several of our co-authors [3] revealed that the negative effects of implemented public health measures have disproportionally affected groups more vulnerable to domestic violence.

Even prior to the pandemic, domestic violence has been an ongoing issue in the US, with >50 % of female homicides occurring during incidents of domestic violence [4]. These incidents of domestic homicide account for nearly 70 deaths per month and over 25 % of all homicides every year [4,5]. According to the American College of Surgeons [6], domestic violence contributes to 30 % of trauma center admissions for women. Unfortunately, these rates of domestic violence may have been exacerbated by the pandemic, as efforts to reduce disease transmission led to social isolation in which individuals (including domestic violence victims) were quarantined away from support systems and were required to combat the unprecedented socioeconomic stressors brought on by COVID-19 [[7], [8], [9]]. These prior authors speculated that forced proximity increases tensions, while also creating new opportunities for violence. In addition, home confinement has had further social and psychodynamic effects on domestic violence survivors as it limits their exposure to the surrounding community, compromising their ability to receive help from others [7,9]. Such isolation also permits fewer opportunities for members of the community to identify and report cases of domestic violence [7].

Furthermore, the rate of firearm purchases during the COVID-19 pandemic is at an all-time high, as there were over 4.3 million estimated firearm purchases between March and July 2020 alone, an 85 % increase in the expected amount calculated from previous years [10,11]. Additionally, between March 2020 and February 2021, there were over 62,000 incidents of firearm violence, a 15 % increase in incidents compared to previous years [12]. This is worrisome as domestic violence and firearm violence are intertwined [13]. In fact, women are up to five times more likely to be killed by their abuser if a firearm is in the home, and firearms have been used in over half of all incidents of domestic homicide [4,13]. Accordingly, the increased rates of firearm purchases and firearm violence during the COVID-19 pandemic may have resulted in a perfect storm of risk factors for increased rates of domestic violence and female homicides during this time [7,14,15]. This study aims to evaluate the impact of the COVID-19 pandemic on patterns and trends of domestic firearm violence (DFV) in the US. We hypothesize the rate of DFV increased in the wake of COVID-19.

## Methods

This study was deemed exempt by the institutional review board and a waiver of consent was granted due to the use of a de-identified national database. All firearm violence data were obtained retrospectively from the Gun Violence Archive (GVA) [16], an independent and online organization that uses automated Internet inquiries to gather incidents of firearm violence across the US from >7500 sources. Data were collected for the years 2018, 2019, 2020, and 2021. Incidents of DFV included in this study were reported by the GVA, which adopts its definition of domestic violence from sources including the Federal Bureau of Investigation, Centers for Disease Control and Prevention, and National Institutes of Health [16].

Annual and monthly DFV counts for 2018, 2019, 2020, and 2021 were collected from the GVA. It is worth noting that, as multiple fatalities or injuries can occur per incident, there are some years in which fatalities are greater than the number of incidents. For the purposes of this study, only incidents involving a male perpetrator and a female victim were included, and female victims of all ages were included with no other exclusion criteria. To control for changes in population across various years, United States Census population reports were used to calculate the rate of firearm violence per 100,000 women in the years 2018–2021 (Supplemental Table 1). Any and all female victims were included in the study for consistent data collection and to perform a statistical comparison between years. Accordingly, given that all victims in the study were females, we used the total number of females for each specific year to comprise the denominator when calculating the rate per 100,000 women (i.e., the number of incidents with a female victim in 2017 divided by the total number of US women in 2017, multiplied by 100,000).

Monthly population data were not available; thus annual population data for each state was used as a denominator to generate a rate of DFV (e.g., the number of incidents divided by the state population) within the GVA per 100,000 women, as monthly population changes in the US are often <0.5 % [17]. To evaluate changes in DFV over time, a one-way ANOVA (with variables defined as categorical) was conducted to compare rates of total average monthly DFV incidents, injuries, and fatalities for each year of the study across the nation. Average monthly DFV incidents were used (rather than average annual incidents) to increase the study's power and granularity for statistical analysis. Shapiro-Wilk tests were employed to investigate if the data were normally distributed prior to ANOVA testing, and it was determined that the data were normally distributed.

Given the potential correlation between legislation and firearm violence, we also performed an analysis where states were categorized based on gun law strength and divided into two groups: the top 25 states with the strongest gun laws and the bottom 25 states with the weakest gun laws [3,5] (Supplemental Table 2). States were separated in this way to investigate the overall association between strong and weak gun laws and rates of DFV. Grouping in this manner was not performed to evaluate any individual state, but rather to examine general trends in DFV based on differing levels of gun law strength. State gun law strength was determined by gun law strength ranking reported by the Gifford's Law Center [18]. First, a one-way ANOVA was performed in which the sub-groups of gun law strength states were analyzed separately to determine individual trends in DFV incidents, injuries, and deaths. Next, a two-way ANOVA was performed to determine if gun law strength (top 25 strongest versus bottom 25 weakest) and year (2018, 2019, 2020, 2021) together had a significant effect on DFV incidents, injuries, and deaths. Values reported for the number of DFV incidents, injuries, and deaths, in strong or weak gun law states represent the average number of all 25 states in that category (strong or weak), and the total population of females (in all 25 states for each category) was used to calculate the average number of incidents, injuries, and fatalities per 100,000 women. The main effect of gun law strength and year were evaluated for significance, as was the effect of the interaction between these variables. Given that each incident of firearm violence results in a number of injuries and fatalities, we further investigated the number of injuries and fatalities per incident of DFV, to identify if gun laws correlated with differences in injuries/fatalities per incident of DFV.

Finally, we also included a bivariate linear regression analysis for the number of injuries and deaths based on gun type (e.g., handgun versus long gun). We also investigated the number of injuries and deaths per incident based on firearm type to assess differences. A *p*-value for all analyses <0.05 was considered statistically significant. All analyses were performed using IBM SPSS Statistics, Version 26 (IBM Corp., Armonk, New York).

## Results

**Fig. 1:**
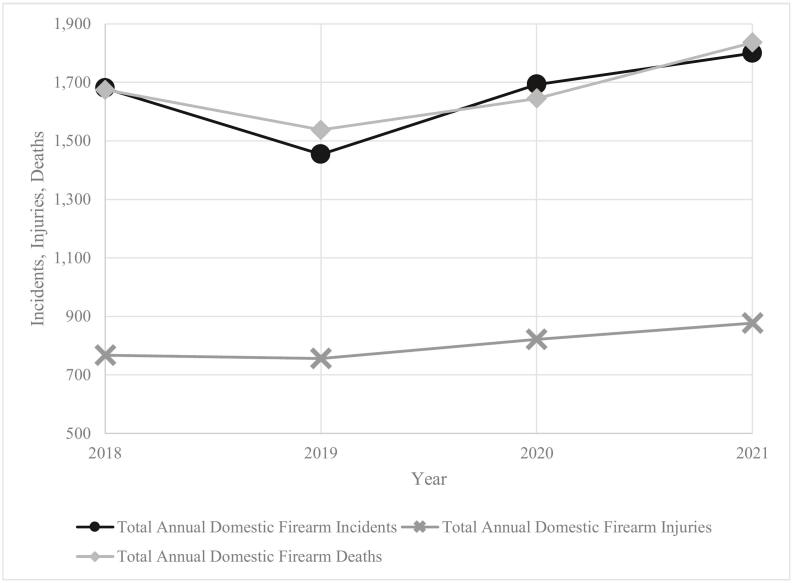
Annual domestic firearm incidents, deaths, and injuries in the United States over time.

There were 1680 DFV shootings in 2018, 1454 in 2019, 1693 in 2020, and 1800 in 2021 (Fig. 1). From 2018 to 2019, there was a 13.4 % decrease in annual incidents of DFV. Contrarily, in 2020, the first year of the pandemic, there was a 16.4 % increase in total incidents compared to 2019. Total annual incidents of DFV then continued to increase by an additional 6.3 % in 2021 from 2020. From the beginning of the studied time period in 2018 to the end of 2021, there was an overall 7.1 % increase in DFV shootings. With one-way ANOVA testing, it was found that the average monthly rate of DFV incidents across the US was highest in 2021 (150 incidents per month) compared to 2018, 2019, and 2020 (*p* = 0.01). There was no difference observed when comparing the average monthly rates of DFV injuries and deaths between each year (all *p* > 0.05) (Table 1).

**Table 1:** One-way ANOVA for average monthly national firearm violence data across all study years.

	2018	2019	2020	2021	p
Firearm incidents (Mean monthly)	140.0 ± 15.1	101.1 ± 49.3	141.1 ± 10.0	150.0 ± 16.6	**<0.01**
Firearm injuries (Mean monthly)	63.9 ± 14.4	63.0 ± 10.3	68.4 ± 11.1	73.1 ± 9.1	0.13
Firearm deaths (Mean monthly)	139.6 ± 35.6	128.2 ± 24.1	137.1 ± 19.9	153.0 ± 24.0	0.16

p values in bold indicate statistical significance (p ≤ 0.05).

### Impact of firearm type and state gun laws on DFV

#### DFV by firearm type

In bivariate linear regression analysis, long gun use more often correlated with a victim's death compared to the use of a handgun (USC B = -0.21, CI: [−0.32 to −0.11], *p* < 0.01) (Table 2). Of DFV in 2018, 22.8 % was attributed to long guns whereas 77.3 % was attributed to handguns. In 2019, 23.41 % of DFV was from long guns and 76.59 % from handguns. DFV from long guns continued to rise to 24.7 % in 2020, with handguns representing 75.3 %. Lastly, in 2021, 21.1 % of DFV was due to long guns with 78.9 % due to handguns.

**Table 2:** Bivariate linear regression for the number of injuries and deaths per incident based on gun type.

	USC B	95 % CI	p
DFV injuries			
Handgun (ref = long gun)	−0.03	(−0.13, 0.06)	0.49
DFV deaths			
Handgun (ref = long gun)	−0.21	(−0.32, −0.11)	**<0.01**

DFV = domestic firearm violence.

#### State gun laws and DFV

In one-way ANOVA testing comparing average monthly rates of DFV incidents, injuries, and deaths for the years 2018 through 2021, it was found that states with stronger gun laws experienced a decrease in DFV incidents from 2018 (63.2 total or 0.06 per 100,000 women) to 2021 (62.1 total incidents or 0.06 per 100,000 women) (*p* = 0.02). However, there was no evidence of a difference in average monthly injuries or fatalities in these stronger gun law states during this same time period.

In contrast, states with weaker gun laws had increased average monthly incidents, fatalities, and injuries over time (all *p* < 0.05). Specifically, there was an increase in average monthly incidents from 2018 (76.8 total incidents or 0.08 per 100,000 women) to 2021 (87.9 total incidents or 0.09 per 100,000 women) (*p* < 0.01), injuries from 2018 (32.9 total incidents or 0.03 per 100,000 women) to 2021 (41.8 total incidents or 0.04 per 100,000 women) (p = 0.02), and an increase in fatalities from 2018 (70.2 total incidents or 0.07 per 100,000 women) to 2021 (88.8 total incidents or 0.09 per 100,000 women) (p < 0.01) (Table 3).

**Table 3:** One-way ANOVA for average monthly national firearm violence data per 100,000 women by strong versus weak gun law states across all study years.

	2018	2019	2020	2021	p
Strong gun law states					
Firearm incidents (mean monthly)	0.06 ± 0.01	0.05 ± 0.01	0.06 ± 0.01	0.06 ± 0.01	**0.015**
Firearm injuries (mean monthly)	0.03 ± 0.01	0.03 ± 0.00	0.03 ± 0.01	0.03 ± 0.01	0.593
Firearm deaths (mean monthly)	0.07 ± 0.02	0.06 ± 0.01	0.06 ± 0.02	0.06 ± 0.02	0.523

Weak gun law states					
Firearm incidents (mean monthly)	0.08 ± 0.01	0.07 ± 0.01	0.08 ± 0.01	0.09 ± 0.01	**<0.001**
Firearm injuries (mean monthly)	0.03 ± 0.01	0.03 ± 0.01	0.04 ± 0.01	0.04 ± 0.01	**0.017**
Firearm deaths (mean monthly)	0.07 ± 0.01	0.07 ± 0.01	0.08 ± 0.01	0.09 ± 0.01	**<0.001**

Upon further analysis of DFV incidents, a two-way ANOVA revealed that gun law strength (F = 112.17, p < 0.01, η2 = 0.01) and year (F = 9.29, p < 0.01, η2 = 0.00) both had a significant effect, with stronger gun laws correlating with fewer DFV incidents. However, in this same model, the interaction between the gun law strength and year variables was non-significant (F = 1.85, *p* = 0.14, η2 = 0.00). Further, Tukey's test for multiple comparisons found that the year 2019 had significantly less DFV incidents compared to other years (all *p* < 0.05).

In terms of DFV injuries, a two-way ANOVA revealed that year (F = 1.116, *p* = 0.341, η2 = 1.918), gun type (F = 0.438, *p* = 0.508, η2 = 0.251), and the interaction between these variables (F = 0.698, *p* = 0.554, η2 = 1.199) did not have a significant association with the number of injuries per DFV incident. Tukey's test for multiple comparisons did not find significant differences between the year and DFV injuries per incident (all *p* < 0.05).

Finally, in terms of DFV deaths, a two-way ANOVA revealed that year (F = 6.851, *p* < 0.001, η2 = 14.482), gun type (F = 17.466, p < 0.001, η2 = 12.307), and the interaction between these variables (F = 3.900, *p* = 0.009, η2 = 8.245) did have a significant association with increased number of deaths per DFV incident. However, Tukey's test for multiple comparisons did not find significant differences between the year and DFV deaths per incident (all p < 0.05).

## Discussion

DFV has remained a national crisis that is almost certainly underreported [19]. This study reveals that the COVID-19 pandemic potentially exacerbated this crisis as incidents increased nationwide, with further demonstration that weaker gun laws are associated with increased DFV, and that long guns are associated with increased lethality in DFV.

Incidents of DFV may have increased during the pandemic for several reasons. Many factors contribute to why domestic violence occurs, with one being a relationship between an aggressor and victim, in which an imbalance of power between them can lead to outbursts of violence as one party, the aggressor, tries to maintain feelings of control [20]. As such, domestic abusers may use the threat of DFV (or commit DFV) to regain control of their environments and partners [21]. The COVID-19 pandemic was a time of significant stress for many individuals, and aggressors may have had an increased feeling of losing control which could have contributed to increased rates of DFV incidents as aggressors lash out at their victims [7,19,21,22]. The national increase in DFV incidents reported in this study may also be partially explained by the increasing rates of firearm violence present throughout the nation during the pandemic, which may be attributed to the large increase in firearm sales that occurred at the beginning of the COVID-19 pandemic, as previously reported by several of our co-authors [3,14,23]. In addition, unsafe firearm storage and the purchase of multiple firearms may have also contributed to increased rates of DFV, as both variables have been associated with an increased risk of firearm violence [10,13,14,23].

Though DFV incidents increased nationwide, states with weaker gun laws were associated with higher rates of incidents, injuries, and fatalities over time, whereas states with stronger gun laws had no such increases from 2018 to 2021. This may be unsurprising, given that prior reports have also found an association between firearm legislation and firearm violence [3,5]. The association between increased DFV in weaker gun law states may be due to increased firearm access and/or less restricted access to firearms by individuals with a history of domestic violence [24]. Regardless, it would appear warranted for weaker gun law states to trial legislation efforts to see if this can curtail DFV.

Furthermore, while it is widely reported that handgun sales surged during this time, trends in the types of guns being sold have reflected an increasing lethality, with long-gun purchases increased by nearly 50 % in 2020 compared to 2019 [3,10]. This fact is particularly concerning given our finding that long guns used in DFV have increased lethality in this vulnerable population [3]. Evidence based efforts to increase primary prevention appears needed nationwide and especially in areas with increased use of long guns [10,25]. Moreover, our reported decrease in DFV in 2019 when compared to 2018 is further corroborated by a 2019 Criminal Victimization Report by the United States Department of Justice [26]. They report the overall rate of violent crimes decreased in 2019, along with domestic violence and sexual crimes, when compared to the previous year [26]. While the exact cause of this is likely multifactorial, the driving factors of DFV are likely similar to causes of violent crime and generalized domestic violence [13]. Future studies investigating specific risk factors for and causes of DFV are needed.

There currently exists a lack of literature regarding DFV, specifically, and this may be in part due to a lack of DFV data as a result of poor standardized data collection and lack of a universal definition. In fact, to the best of our knowledge, this piece is the first study to utilize a nationally aggregated firearm violence database to investigate DFV over time. Furthermore, in general, domestic violence across the US and worldwide is vastly underreported due to a fear of retaliation, shame from the incident, and/or a fear that the victim will not be believed, which may especially skew the reporting of non-fatal incidents [21]. Prior authors have attempted to extrapolate data on this topic [13,19,21]. However, statistical methods and prior data sets utilized are not ideal. This current national analysis utilizing the GVA provides a more comprehensive evaluation of this topic although still undoubtedly underreports the incidence of domestic firearm violence.

This study has multiple limitations including those inherent to its retrospective design and use of the GVA database. This includes misclassification errors and unreported incidents. The GVA works by reporting automated internet inquiries. Therefore, regions of the United States in which DFV victims have less access to police departments or other resources capable of reporting incidents of domestic violence are likely to suffer from underreporting of incidents. Moreover, misclassification errors may have occurred, as, outside of gun laws, no other consideration into specific state, county, or city policies or characteristics were taken into account as this was felt to be not plausible given the complexity of city/county/state firearm legislation. As incidents were found using *domestic violence*, as a search rule, incidents are to be categorized only if they fall under the definition of domestic violence provided by the GVA. This may have led to some incidents of domestic violence to have gone unreported in this study as they may have been categorized by the GVA under another definition. Additionally, though there have been few comprehensive studies conducted to confirm the reliability, reproducibility, or validity of the GVA database, a recent study by Gobaud et al. revealed that the GVA is generally an appropriate model for firearm violence research. Despite these limitations, the GVA is one of the most comprehensive databases on this topic that has been utilized in numerous studies and by news sources [3,[27], [28], [29], [30]]. Furthermore, the methods of collection by the GVA have changed over time and vary depending on the location (e.g., increased coverage of urban compared to rural locations). In addition, the authors believe the GVA should transparently disclose its data acquisition methods to foster improved understanding of its database as a source utilized by media and researchers alike.

Lastly, to address the epidemic of DFV there should be a continued emphasis on evidence-based practices, such as safe firearm storage, primary prevention for high-risk populations, increased public awareness of the pervasiveness of DFV, and the implementation of legislation that enhances firearm restrictions for perpetrators of domestic violence [13,24,29,30].

## Conclusion

This national analysis found that there was an overall increase in DFV nationwide during the COVID-19 pandemic. Furthermore, the state in which the violence occurred and the use of a long gun led to differing rates of victim injury and death. As states with weaker gun laws had increased incidents, fatalities, and injuries, policy-level interventions may help mitigate the increased harm done by DFV in these states. In addition, primary prevention efforts, especially related to long guns which had increased lethality in this study, appear warranted.

## Funding sources

NA

## Ethics approval

This study was deemed exempt by the institutional review board and a waiver of consent was granted due to the use of a de-identified national database.

## CRediT authorship contribution statement

**Jonathan Shipley:** Conceptualization, Data curation, Formal analysis, Resources, Validation, Writing – original draft. **Megan Donnelly:** Writing – original draft, Writing – review & editing. **Catherine Kuza:** Supervision. **Areg Grigorian:** Supervision, Writing – original draft. **Lourdes Swentek:** Conceptualization, Writing – original draft. **Theresa Chin:** Methodology, Resources, Supervision. **Nolan Brown:** Data curation, Investigation. **Ninh Nguyen:** Investigation. **Jeffry Nahmias:** Methodology, Resources, Supervision, Writing – original draft, Writing – review & editing.

## Declaration of competing interest

None.

## Appendix A Supplementary data

Supplementary tables Image 1
