# Supplementary material for: Domestic firearm violence against women (2018–2021)
Source: Surg Open Sci. 2024 Jan 15;17:75–9. doi: 10.1016/j.sopen.2024.01.010 (PMC10828568; doi:10.1016/j.sopen.2024.01.010)
Supplement: Supplementary file 1 — Supplementary tables [file mmc1.docx]

**Supplemental Table 1.** Population of Female Individuals by State for 2018, 2019, 2020, and 2021**.**

|  | **2018** | **2019** | **2020** | **2021** |
| --- | --- | --- | --- | --- |
| Alabama | 2524361 | 2533668 | 2582589 | 2591778 |
| Alaska | 351692 | 350127 | 348498 | 348469 |
| Arizona | 3600640 | 3661420 | 3593335 | 3642602 |
| Arkansas | 1531624 | 1536188 | 1525393 | 1532713 |
| California | 19843136 | 19867369 | 19747029 | 19620646 |
| Colorado | 2825526 | 2857388 | 2854321 | 2867419 |
| Connecticut | 1829397 | 1825870 | 1832774 | 1836010 |
| Delaware | 498641 | 502971 | 509677 | 516048 |
| Florida | 10858944 | 10980681 | 10963711 | 11070771 |
| Georgia | 5399300 | 5456999 | 5486425 | 5529805 |
| Hawaii | 709302 | 707832 | 720606 | 716220 |
| Idaho | 873205 | 891137 | 916163 | 942307 |
| Illinois | 6471727 | 6445263 | 6463736 | 6406387 |
| Indiana | 3394211 | 3411859 | 3418402 | 3428315 |
| Iowa | 1581655 | 1583995 | 1590874 | 1591695 |
| Kansas | 1460588 | 1461617 | 1465014 | 1464091 |
| Kentucky | 2263603 | 2266644 | 2271992 | 2275505 |
| Louisiana | 2385448 | 2381744 | 2370694 | 2358856 |
| Maine | 683374 | 686100 | 690551 | 695754 |
| Maryland | 3111203 | 3116815 | 3164642 | 3162705 |
| Massachusetts | 3542925 | 3546935 | 3591124 | 3572631 |
| Michigan | 5067862 | 5068329 | 5078919 | 5070232 |
| Minnesota | 2813822 | 2829821 | 2846606 | 2846977 |
| Mississippi | 1535497 | 1533857 | 1516839 | 1514629 |
| Missouri | 3116702 | 3124766 | 3116648 | 3123522 |
| Montana | 526813 | 530712 | 536177 | 545001 |
| Nebraska | 964240 | 967959 | 976215 | 976900 |
| Nevada | 1508820 | 1535329 | 1544659 | 1559894 |
| New Hampshire | 682692 | 685918 | 690634 | 695860 |
| New Jersey | 4545338 | 4541603 | 4712363 | 4705188 |
| New Mexico | 1059397 | 1057201 | 1063737 | 1063180 |
| New York | 10046133 | 10005715 | 10304981 | 10140611 |
| North Carolina | 5331288 | 5387820 | 5342825 | 5394907 |
| North Dakota | 370046 | 372030 | 377973 | 376539 |
| Ohio | 5953904 | 5958538 | 5973753 | 5967819 |
| Oklahoma | 1987969 | 1996313 | 1989232 | 2002339 |
| Oregon | 2108799 | 2127010 | 2126770 | 2129317 |
| Pennsylvania | 6529381 | 6527628 | 6579380 | 6564246 |
| Rhode Island | 543427 | 543686 | 559025 | 558649 |
| South Carolina | 2620391 | 2655575 | 2633756 | 2666990 |
| South Dakota | 435205 | 437902 | 436711 | 440534 |
| Tennessee | 3466921 | 3496935 | 3525719 | 3555217 |
| Texas | 14409351 | 14593179 | 14631793 | 14791971 |
| Utah | 1565310 | 1591041 | 1620532 | 1647906 |
| Vermont | 316079 | 315673 | 322962 | 324720 |
| Virginia | 4318025 | 4335262 | 4359066 | 4365073 |
| Washington | 3758918 | 3802910 | 3831057 | 3840425 |
| West Virginia | 910982 | 904377 | 897367 | 893861 |
| Wisconsin | 2918181 | 2925225 | 2940452 | 2942034 |
| Wyoming | 283325 | 284029 | 281448 | 282560 |

**Supplemental Table 2.** Jurisdictions in the United States Classified as Strong Versus Weak Gun Law States in the Most Recent Study Year (2021)

| **State** | **Strong Versus Weak Gun Law State** | **Gun Law Strength** | **Gun Death Rate Rank** | **Gun Deaths Per 100 K** |
| --- | --- | --- | --- | --- |
| California | Strong | 1 | 43 | 9 |
| New Jersey | Strong | 2 | 48 | 5.2 |
| Connecticut | Strong | 3 | 45 | 6.7 |
| New York | Strong | 4 | 47 | 5.4 |
| Hawaii | Strong | 5 | 49 | 4.8 |
| Massachusetts | Strong | 6 | 50 | 3.4 |
| Maryland | Strong | 7 | 29 | 15.2 |
| Illinois | Strong | 8 | 26 | 16.1 |
| Rhode Island | Strong | 9 | 46 | 5.6 |
| Washington | Strong | 10 | 39 | 11.2 |
| Oregon | Strong | 11 | 30 | 14.9 |
| Virginia | Strong | 12 | 32 | 14.3 |
| Delaware | Strong | 13 | 23 | 16.6 |
| Colorado | Strong | 14 | 18 | 17.8 |
| Pennsylvania | Strong | 15 | 31 | 14.8 |
| Michigan | Strong | 16 | 28 | 15.4 |
| Nevada | Strong | 17 | 15 | 19.8 |
| Minnesota | Strong | 18 | 42 | 10 |
| New Mexico | Strong | 19 | 3 | 27.8 |
| Nebraska | Strong | 20 | 41 | 10.3 |
| North Carolina | Strong | 21 | 21 | 17.3 |
| Vermont | Strong | 22 | 38 | 11.9 |
| Florida | Strong | 23 | 34 | 14.1 |
| Wisconsin | Strong | 24 | 36 | 13.5 |
| Ohio | Strong | 25 | 24 | 16.5 |
| New Hampshire | Weak | 26 | 44 | 8.3 |
| Maine | Weak | 27 | 37 | 12.6 |
| South Carolina | Weak | 28 | 11 | 22.4 |
| Indiana | Weak | 29 | 16 | 18.4 |
| Texas | Weak | 30 | 27 | 15.6 |
| Utah | Weak | 30 | 35 | 13.9 |
| Iowa | Weak | 32 | 40 | 11.2 |
| Louisiana | Weak | 32 | 2 | 29.1 |
| Georgia | Weak | 34 | 14 | 20.3 |
| Oklahoma | Weak | 34 | 12 | 21.2 |
| West Virginia | Weak | 36 | 19 | 17.3 |
| North Dakota | Weak | 37 | 22 | 16.8 |
| Alabama | Weak | 38 | 4 | 26.4 |
| Tennessee | Weak | 38 | 10 | 22.8 |
| Montana | Weak | 40 | 7 | 25.1 |
| Alaska | Weak | 41 | 6 | 25.2 |
| Arizona | Weak | 42 | 17 | 18.3 |
| Kentucky | Weak | 43 | 13 | 21.1 |
| South Dakota | Weak | 44 | 33 | 14.3 |
| Kansas | Weak | 45 | 20 | 17.3 |
| Mississippi | Weak | 45 | 1 | 33.9 |
| Missouri | Weak | 47 | 9 | 23.2 |
| Idaho | Weak | 48 | 25 | 16.3 |
| Wyoming | Weak | 49 | 5 | 26.1 |
| Arkansas | Weak | 50 | 8 | 23.3 |
